# Laboratory biomarkers associated with COVID-19 mortality among inpatients in a Peruvian referral hospital

**DOI:** 10.1016/j.heliyon.2024.e27251

**Published:** 2024-02-29

**Authors:** Stephanie Montero, Jorge L. Maguiña, Percy Soto-Becerra, Virgilio E. Failoc-Rojas, Jorge Chira-Sosa, Moisés Apolaya-Segura, Cristian Díaz-Vélez, Stalin Tello-Vera

**Affiliations:** a Instituto de Evaluación de Tecnologías en Salud e Investigación - IETSI, ESSALUD, Lima, Peru; b Facultad de Ciencias de la Salud, Universidad Científica del Sur, Lima, Peru; c Universidad Continental, Huancayo, Peru; d Unidad de Investigación para la Generación y Síntesis de Evidencias en Salud, Universidad San Ignacio de Loyola, Lima, Peru; e Laboratorio de Biología Molecular, Citometría de flujo y Citogenética, Hospital Nacional Almanzor Aguinaga Asenjo, ESSALUD, Chiclayo, Peru; f Facultad de Medicina Humana, Universidad Privada Antenor Orrego, Trujillo, Peru; g Escuela de Medicina, Universidad César Vallejo, Trujillo, Peru

**Keywords:** COVID-19, SARS-CoV-2, Biochemical, Hematological, Biomarkers

## Abstract

**Aim:**

To evaluate the biochemical and hematological markers associated with the risk of death due to COVID-19 in a clinical cohort with a severe clinical profile.

**Methods:**

A retrospective study was conducted among 215 anonymized inpatient records from the Hospital Nacional Almanzor Aguinaga Asenjo, Peru, between April and June 2020. The association between biomarkers and death due to COVID-19 was assessed using Cox regression, with a multivariable modeling of 1) biochemical and 2) hematological markers. Kaplan-Meier analyses and time-dependent receiver operating characteristic curves were calculated for each associated biomarker (p < 0.05).

**Results:**

Data analysis of 215 inpatient records revealed an overall mortality rate of 51.30% (95% CI 44.70–58.50), a mean age of 63.90 ± 14.10 years, and a median oxygen saturation of 88% (interquartile range 82–92%). The best-fitted biochemical model included higher levels of C-reactive protein (CRP), D-dimer, fibrinogen, urea, and lactate dehydrogenase. Similarly, the best-fitted hematological model included higher absolute neutrophil and prothrombin time, and lower absolute platelet counts. The best area under the curve values in both models were found to be CRP and D-dimer values (>0.74) and the absolute neutrophil count (0.63).

**Conclusions:**

Some specific biochemical markers outperformed hematological markers. Evaluated hematological counts analyzed in multivariable models proved to be better markers and could be useful to discriminate COVID-19 patients at high risk of death.

## 1 Background

The COVID-19 emergency has resulted in multi-level shortages of resources, leading to the collapse of health systems worldwide, notably in Latin America [[1], [2], [3]]. Peru has the highest standardized mortality rate globally, with 6552 deaths per 1 million inhabitants [4]. The lack of access to hospital care opportunities, the widespread use of therapies lacking scientific evidence, and self-medication contributed to poor outcomes in many patients [5,6].

SARS-CoV-2 infection is mediated by the interaction between the receptor-binding domain of the viral Spike protein and the human angiotensin-converting enzyme 2 (ACE-2) expressed in pneumocytes [7]. Infection induces the overproduction of proinflammatory, leading to a cytokine storm that can result in acute respiratory distress syndrome and multiple organ dysfunction [8,9]. The cascade of immunological and metabolic reactions produces key biomarkers that are over- and under-expressed during the evolution of COVID-19 within the host.

Several biomarkers associated with the severity and mortality of COVID-19 have been reported, mainly in single-site studies. Inflammatory markers include interleukins (IL) 1b/6/8, C-reactive protein (CRP), ferritin, creatinine kinase (CK), and the neutrophil/lymphocyte ratio (NLR), while coagulation biomarkers include D-dimer, fibrinogen, and platelet count (thrombocytopenia). Indicators of cardiac compromise include troponin and lactate dehydrogenase (LDH). Predictors of bacterial co-infection include the lymphocyte count (lymphopenia) and procalcitonin, and liver damage is assessed by alanine (ALT) and aspartate transaminases (AST) [8,10,11].

For clinical decision-making, it is recommended to use mainly biochemical markers such as elevated levels of D-dimer, CRP, LDH, troponin, ferritin, and CK, as well as a decrease in absolute lymphocyte counts and an increase of NLR, as indicators of COVID-19 severity [[11], [12], [13], [14], [15], [16]]. However, further studies are needed to determine cut-off points in populations with severe clinical profiles and to confirm their real diagnostic power in larger populations. Despite the usefulness of these biomarkers, their determination is not available in health centers with low complexity levels. Therefore, it is fundamental to use accessible laboratory biomarkers to classify patients early according to their prognosis.

In addition to biochemical markers, the complete blood count (CBC) provides valuable information related to inflammatory, infectious, and coagulation factors [8,17]. Hematological markers are routinely assessed upon admission and during the follow-up of all inpatients (moderate or severe) due to their suitability. Despite massive vaccination decelerating the spread of COVID-19, there are still primary care centers attending COVID-19 cases operating under precarious conditions. Therefore, it is essential to use accessible resources to support clinical decision-making and for the timely allocation of resources to patients at risk. Understanding the role of biomarkers as a clinical decision support tool will allow the early classification of COVID-19 cases requiring specific care according to their prognostic risk. The present study aimed to evaluate the prognostic value of biochemical and hematological markers in patients with severe clinical symptoms associated with mortality due to COVID-19.

## 2 Methods

### 2.1 Study design

A retrospective cohort study was conducted at Hospital Nacional Almanzor Aguinaga Asenjo (HNAAA), which is part of the Seguro Social de Salud (ESSALUD), in the northern Peruvian city of Lambayeque. HNAAA has 404 hospitalization beds and 38 intensive care unit (ICU) beds to normally serve 660,506 insured persons. However, during the peaks of the COVID-19 pandemic, this hospital attended to any patient in need of hospital care in northern Peru, encompassing 1,197,260 inhabitants.

### 2.2 Population

Data from anonymized records of patients admitted to the hospital between April and June 2020 were evaluated. The study population consisted of inpatients admitted during the first wave of COVID-19 who manifested respiratory distress, oxygen saturation (SpO2) <93%, and a partial pressure of arterial oxygen/oxygen concentration ≤300 mmHg [18]. Clinically compatible COVID-19 cases were confirmed using a rapid anti-SARS-CoV-2 antibody serological test (RT-PCR tests were not available) following national criteria [19]. All patients were followed throughout the entire course of their progression until hospital discharge (death or survival), and none were referred to another healthcare facility.

### 2.3 Data

Demographic and clinical data, as well as patient disposition (discharge or death), were extracted from medical records and directly digitized into a database. The laboratory parameters included in the study corresponded to the first results evaluated in hospitalized patients during the admission period, presenting severe or critical COVID-19.

### 2.4 Variables

Death due to COVID-19 was the primary outcome, and the date of death was recorded by the attending physician. Sex and comorbidities, as well as the presence of granulocyte precursors and atypical lymphocytes in peripheral blood, were considered as categorical covariates. Age, disease duration, length of hospitalization, SpO2, complete blood count (hemoglobin, hematocrit, platelets, plateletcrit, leukocytes, lymphocytes, neutrophils, mean platelet volume, platelet distribution width, platelet large cell ratio), prothrombin time, partial thromboplastin time activated, International Normalized Ratio (INR), and biochemical markers (D-dimer, ferritin, LDH, urea, CRP, creatinine, ALT, AST, glucose) were analyzed as continuous variables. Reference ranges are found in **Additional file 1**. All laboratory biomarkers were evaluated using the first result at hospitalization.

### 2.5 Statistical analysis

Continuous variables were summarized as mean and standard deviation (SD) or median and interquartile range (IQR). Categorical covariates were summarized as absolute and relative frequencies. Bivariate analysis was performed using the Chi-square, Fisher exact, Student's-t or median test, depending on the data. We addressed missing data with multiple imputations (**Additional file 2**). We also plotted linearity between all biomarkers and the outcome (**Additional file 3**).

Patient survival was also estimated by comparing the tertiles of biomarkers using Kaplan-Meier. Log-rank test was calculated to determine global differences between survival curves without crossing patterns, and the Flemington-Harrington test for the crossed curves, which evaluates late differences (p < 0.001) [20].

Non-linear biomarkers were transformed into restrictive cubic splines (RCS) with four knots, and significant RCS variables, determined by the Wald test (p < 0.05), were then included for multivariable modelling. Multivariable models were generated using Cox regression and the nested model method with backward selection. A model composed of biochemical markers was built using all the variables from the database. Likewise, three models (A, B, and C) composed only of hematological markers (excluding biochemical markers) were built to evaluate indicators available in healthcare centers with low complexity levels. We modeled: A) all hematological variables, B) variables excluding relative hematological values, and C) complete blood count values. The most parsimonious models were selected using the Wald test (p < 0.05) for a pool analysis of imputed datasets.

Finally, the individual performance of explanatory biomarkers was evaluated by a time-dependent receiver operating curves (ROC) analysis (30 days), estimating the area under the curve (AUC) for each biomarker, sensitivity, and specificity for an optimal cut-off point according to the Youden index, as implemented in the package sttroccurve [21]. Also, positive and negative predictive values were estimated. All analyses were conducted using Stata software v.17 (Stata Corp. College Station, TX).

### 2.6 Ethics

The study protocol received approval from the Ethics and Research Committee of the Red Prestacional Lambayeque - Hospital Nacional Almanzor Aguinaga Asenjo, determination N° 026-CIEI-RPLAMB. Since the analysis was conducted with anonymized data, the Ethics Committee provided an exemption from the informed consent procedure.

## 3 Results

### 3.1 Epidemiological characteristics

The data of 215 patients were analyzed, identifying the proportion of death at 51.60% (95% CI 44.70–58.50), and the overall fatality rate at day 30 in 66.60% (95% CI 57.70–75.40). The mean age was 63.90 years (SD 14.14) and the median SpO2 determined at admission was 88%. (Table 1).

**Table 1:** Characteristics and biochemical markers of the study population.

	Total n = 215 (%)	Survivors n = 104	Deaths n = 111	P^b^
Age (years)	63.90 ± 14.14	60.06 ± 14.49	67.50 ± 12.86	**<0.001**
Sex				
Women	61 (28.37)	32 (52.46)	29 (47.54)	0.450
Men	154 (71.63)	72 (46.75)	82 (53.25)	
Disease duration (days)^a^	9.06 ± 4.06	8.55 ± 3.76	9.54 ± 4.30	0.078
Length of hospital stay (days)	11 [6–18]	14 [8–21]	8 [5–14]	**<0.001**
Comorbidities				
Obesity	21 (9.77)	7 (33.33)	14 (66.67)	0.147
Diabetes mellitus	51 (23.72)	22 (43.14)	29 (56.86)	0.392
Hypertension	82 (38.14)	32 (39.02)	50 (60.98)	**0.031**
Chronic kidney disease	21 (9.77)	4 (19.05)	17 (80.95)	**0.005**
Other pulmonary diseases	21 (9.77)	12 (57.14)	9 (42.86)	0.397
N° comorbidities				
0	78 (36.28)	40 (51.28)	38 (48.72)	0.057
1	89 (41.40)	48 (53.93)	41 (46.07)	
≥2	48 (22.33)	16 (33.33)	32 (66.67)	
Oxygen saturation (%)	88 [82–92]	90 [88–94]	85 [79–90]	**<0.001**
Fibrinogen (mg/dl)^a^	519 [400–654]	488.5 [372–640]	567 [450–700]	0.210
D-dimer (ug/ml)^a^	2.30 [1.36–4.56]	1.40 [0.90–2.42]	4 [2.20–5]	**<0.001**
Lactate dehydrogenase (U/L)^a^	350 [256–467]	273 [225–350]	439.50 [345–526]	**<0.001**
C-reactive protein (mg/dl)^a^	10.70 [4.20–17.7]	5 [1.50–11]	15.80 [10.20–22.30]	**<0.001**
Urea (mg/dl)^a^	40.85 [30.90–56.80]	35.90 [27.60–44.80]	49.60 [33–66.40]	**<0.001**
Ferritin (mg/dl)^a^	1330 [800−2000]	850 [689−1300]	1952.50 [1409–2000]	**<0.001**
Aspartate aminotransferase (U/L)^a^	32.65 [21.10–51]	32.70 [21–51]	32.60 [22–51]	1.000
Alanine aminotransferase (U/L)^a^	41 [23–69]	45 [24–81.30]	37 [22–62]	0.274
Glucose level (mg/dl)	129 [105–171]	123.50 [101.50–166]	135 [106–175]	0.303
Creatinine level (mg/dl)^a^	0.69 [0.55–0.92]	0.66 [0.51–0.82]	0.73 [0.58–1.02]	0.109

Data is presented as n (%), mean ± standard deviation (SD), and median [IQR].

a Missing data was identified in the following variables: length of the disease = 2, SpO2 = 1, D-dimer = 6, fibrinogen = 9, LDH = 2, CRP = 13, urea = 1, ferritin = 24, ALT = 1, AST = 1, creatinine = 5.

b Bivariate analysis was performed using Student's-t and Median test for continuous variables with normal and non-normal distribution, respectively, and Chi-square for categorical variables.

### 3.2 Laboratory findings

Overall, the study population presented a severe clinical picture represented by leukocytosis, neutrophilia and severe lymphopenia. Coagulation markers such as platelet count, plateletcrit and PT ranged between normal values. However, the overall median fibrinogen levels exceeded references ranges (>500 mg/dl). Among biomarkers associated with COVID-19 severity, all the patients presented elevated CRP levels (>0.5 mg/dl), indicating the presence of an advanced inflammatory process at the time of admission (Table 1, Table 2).

**Table 2:** Hematological count, presence of granulocyte precursors and atypical lymphocytes on peripheral blood.

	Total n = 215 (%)	Survivors n = 104	Deaths n = 111	P^b^
Hemoglobin level (g/dL)	13.28 ± 1.89	13.27 ± 1.83	13.28 ± 1.95	0.955
Hematocrit %	40 [37–43]	40 [37–42.85]	40 [37–43]	0.951
White blood cell count x 10^3^/μl	11.71 [8.50–16.18]	9.79 [7.41–12.69]	13.90 [10.97–19.12]	**<0.001**
Relative lymphocyte count (%)	6 [4–10]	7 [4–12]	5 [3–8]	**0.006**
Absolute lymphocyte count x 10^3^/μl	0.71 [0.45–1.12]	0.69 [0.44–1.13]	0.71 [0.45–1.11]	0.538
Relative neutrophil count (%)	89 [84–93]	87 [80–93]	91 [86–94]	**0.003**
Absolute neutrophil count x 10^3^/μl	10.30 [7.30–14.49]	8.42 [6.29–11.61]	12.78 [9.39–16.44]	**<0.001**
Neutrophil/Lymphocyte ratio	14.50 [8.40–23.51]	12.79 [7.09–23.12]	15.35 [9.89–30.37]	**0.012**
Platelet count x 10^3^/μl	322.11 ± 120.10	349.54 ± 124.72	296.41 ± 110.07	**0.001**
Mean platelet volume	9.67 ± 1.10	9.68 ± 1.16	9.67 ± 1.04	0.935
Platelet distribution width	16.30 [16.10–16.60]	16.30 [16–16.50]	16.40 [16.10–16.60]	0.183
Plaquetocrit %	0.30 ± 0.10	0.33 ± 0.11	0.28 ± 0.10	**<0.001**
Platelet large cell ratio %	24.33 ± 7.07	24.08 ± 7.31	24.56 ± 6.87	0.619
Prothrombin time^a^	11.10 [10.40–12.10]	10.85 [10.20–11.50]	11.45 [10.60–12.40]	**0.006**
Partial thromboplastin time activated^a^	1 [0.90–29.70]	1 [0.90–29.50]	1 [0.90–29.70]	0.396
International normalized ratio for coagulation factors^a^	26 [1–32]	24.50 [0.90–30]	27.60 [1–34.40]	0.150
Atypical lymphocytes	132 (61.68)	60 (45.45)	73 (54.55)	0.243
Promyelocytes	1 (0.47)	0 (0)	1 (100)	NA
Myelocytes	48 (22.43)	26 (54.17)	22 (45.83)	0.381
Metamyelocytes	54 (25.23)	28 (51.85)	26 (48.15)	0.580
Band form neutrophils	123 (57.75)	53 (43.09)	70 (56.91)	0.050

Data is presented as n (%), mean ± standard deviation (SD), and median [IQR].

a Missing data was identified in the following variables: PT = 1, aPTT = 1, INR = 2, promyelocytes = 1, myelocytes = 1, metamyelocytes = 1, band form neutrophils = 2.

b Bivariate analysis was performed using Student's-t and Median test for continuous variables with normal and non-normal distribution, respectively, and Chi-square for categorical variables.

### 3.3 Biomarkers and mortality by COVID-19

Leukocytosis and neutrophilia at admission were significantly higher among fatal cases (p < 0.001). Additionally, the NLR was higher among fatal cases, with a median of 15.43 (p = 0.01). Overall, patients with a poor prognosis had lower platelet counts, lower plateletcrit, and a longer PT than those who survived (p < 0.05) (Table 1). Coagulation alterations were higher among the fatal cases, presenting three times the median of survivors (both above the normal value < 0.50 μg/ml). Besides, CRP, ferritin, urea, and LDH values were severely elevated in non-survivors. In the bivariate analysis of survival vs. death, D-dimer, CRP, ferritin, urea and, LDH showed statistical significance p < 0.001 (Table 1), contrary to what was identified in granulocyte precursors and atypical lymphocytes in peripheral blood (Table 2).

### 3.4 Kaplan-Meier survival analysis

**Fig. 1:**
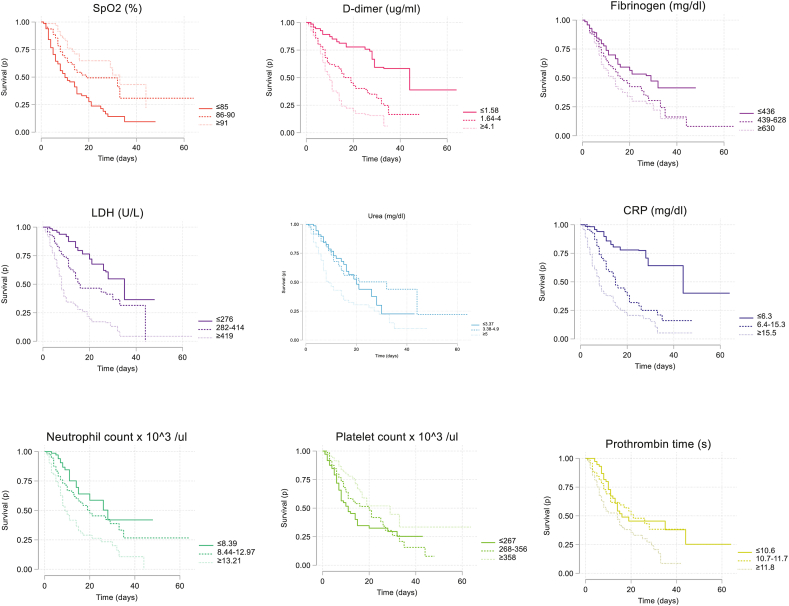
Kaplan-Meier survival analysis for explanatory biomarkers categorized in tertiles. Log-rank test was calculated to determine global differences between survival curves without crossing patterns (SpO2, D-dimer, LDH, CRP; p < 0.001), and the Flemington-Harrington test for the crossed curves, which evaluates late differences (fibrinogen, urea, neutrophil count, platelet count and prothrombin time; p < 0.001).

Differences in the mortality rate were observed along survival functions for SpO2 and tertile biomarkers. As expected, the survival of patients from the lowest SpO2 tertile (≤55%) was lower than the high (≥91%) and medium (56–90%) tertiles. Regarding the explanatory biomarkers, survival was much higher in patients with lower D-dimer and fibrinogen, and higher CRP. The survival curves for the other markers did not show sustained trends at each tertile over time (Fig. 1).

### 3.5 Non-adjusted and adjusted Cox regression models

For each additional year in age at hospital admission, the risk of mortality increased by 2% (p = 0.05). Moreover, a high SpO2 was associated with greater protection against death (3% less risk for each additional unit in the percentage of SpO2, p < 0.001). Hematological markers, including the absolute leukocyte count, relative and absolute lymphocyte count, relative and absolute neutrophil count (8% greater risk for each 10^3^ cell increase), platelets, plateletcrit, and PT, were significantly associated with mortality (p < 0.05). Likewise, biochemical markers such as D-dimer (14% higher risk for each increase in one unit), fibrinogen, LDH, CRP, urea, ferritin, and creatinine (p < 0.005) were also associated with mortality (Table 3).

**Table 3:** Non-adjusted analysis of biomarkers associated with mortality by COVID-19.

	COVID-19 mortality		
	HR	95% CI	p
Age (years)	**1.02**	**(1.01**–**1.03)**	**0.005**
Sex			
Women	Ref		
Men	1.00	(0.65–1.53)	0.997
Disease duration (days)^a^	1.03	(0.99–1.07)	0.122
Comorbidities			
Obesity	1.65	(0.94–2.90)	0.081
Diabetes mellitus	1.01	(0.66–1.54)	0.968
Hypertension	1.30	(0.89–1.90)	0.169
Chronic kidney disease	**1.97**	**(1.17**–**3.31)**	**0.010**
Other pulmonary disease	0.87	(0.44–1.73)	0.700
N° comorbidities			
0	Ref		
1	0.93	(0.60–1.45)	0.757
≥2	1.23	(0.77–1.99)	0.387
Oxygen saturation (%)^a^	**0.97**	**(0.95**–**0.98)**	**<0.001**
Hemoglobin (g/dL)	0.98	(0.90–1.08)	0.743
Hematocrit (%)	1.00	(0.97–1.04)	0.885
White blood cell count x 10^3^/μl	**1.07**	**(1.04**–**1.11)**	**<0.001**
Relative lymphocyte count (%)	**0.94**	**(0.89**–**0.98)**	**0.004**
Absolute lymphocyte count x 10^3^/μl	1.07	(0.75–1.51)	0.723
Relative neutrophil count (%)	**1.04**	**(1.01**–**1.07)**	**0.008**
Absolute neutrophil count x 10^3^/μl	**1.08**	**(1.05**–**1.12)**	**<0.001**
Neutrophil/Lymphocyte ratio	1.01	(0.997–1.02)	0.160
Platelet count x 10^3^/μl^b^	**0.98**	**(0.96**–**0.99)**	**0.003**
Mean platelet volume	1.00	(0.84–1.20)	0.981
Platelet distribution width	0.99	(0.86–1.14)	0.923
Plaquetocrit %	**0.06**	**(0.01**–**0.34)**	**0.002**
Platelet large cell ratio	1.00	(0.98–1.03)	0.728
Prothrombin time^a^	**1.03**	**(1.001**–**1.06)**	**0.044**
Partial thromboplastin time activated^a^	1.00	(0.99–1.02)	0.464
International Normalized Ratio for coagulation factors^a^	1.00	(0.99–1.01)	0.536
Fibrinogen (mg/dl)^a^^,^^b^	**1.02**	**(1.01**–**1.03)**	**0.002**
D-dimer (ug/ml)^a^	**1.14**	**(1.09**–**1.19)**	**<0.001**
Lactate dehydrogenase(U/L)^a^^,^^b^	**1.02**	**(1.02**–**1.03)**	**<0.001**
C-reactive protein (mg/dl)^a^	**1.06**	**(1.04**–**1.07)**	**<0.001**
Urea (mg/dl)^a^^,^^b^	**1.07**	**(1.04**–**1.10)**	**<0.001**
Ferritin (mg/dl)^a^^,^^b^	**1.01**	**(1.01**–**1.02)**	**<0.001**
Aspartate aminotransferase (U/L)^a^	1.00	(0.996–1.01)	0.760
Alanine aminotransferase (U/L)^a^	1.00	(0.99–1.000)	0.067
Glucose (mg/dl)	1.00	(0.999–1.002)	0.243
Creatinine (mg/dl)^a^	**1.06**	**(1.002**–**1.12)**	**0.040**

HR, hazard ratio; CI, confidence interval.

The non-adjusted analysis was conducted using Cox regression, which included imputed datasets. Values in bold indicate significant associations (p < 0.05).

a Data with missing values.

b Variables scaled/10 to better interpret the HR.

**Fig. 2:**
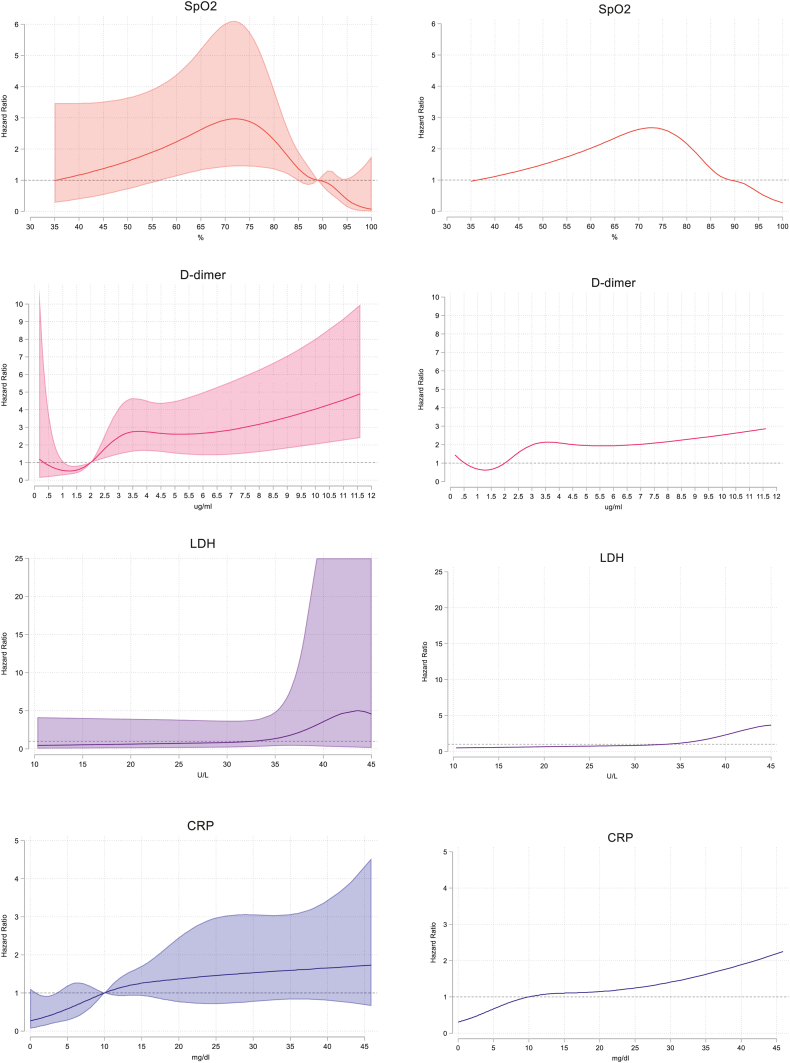
Non-linear associations between biomarkers and death by COVID-19. Restricted cubic spline models fitted for Cox proportional hazards models with four knots for three significant non-linear associations from the principal analysis. We used as reference values the cut-offs estimated for ROC analysis, on the left, plots generated with case-complete data, and on the right side, plots generated with imputed datasets. Only SpO2 and D-dimer represent a reliable continuous relationship with HR estimations. Lower SpO2 % represents a higher risk, which is dynamic depending on the sample size (values < 75% lack of precision). On the contrary, higher D-dimer values are related dynamically to death by COVID-19 (values >9ug/ml risk of lacking precision).

Several adjusted regression models were built with the incorporation of variables following technical criteria. One model composed of the most explanatory biochemical markers for mortality by COVID-19 included: low SpO2, and high levels of D-dimer, fibrinogen, CRP, urea, LDH and platelet count (Table 4a). In addition, models were built using only hematological markers, resulting in three explanatory models composed of hematological markers associated with risk for death, accompanied by low SpO2. Explanatory covariates of mortality by COVID-19 included: A) and B) both models converged in selecting high number of neutrophils, low platelet count and longer PT; and C) high number of neutrophils and low platelet count. (Table 4b). Moreover, the dynamic relationship between RCS of SpO2, D-dimer, CRP and LDH are shown in Fig. 2.

**Table 4a:** Adjusted analysis of biochemical markers associated with COVID-19 mortality.

	COVID-19 mortality – biochemical markers					
	HR	95% CI	p	aHR	95% CI	p
Oxygen saturation (%)	0.97	(0.95–0.98)	**<0.001**	b		
D-dimer (ug/ml)	1.14	(1.09–1.19)	**<0.001**	b		
Fibrinogen (mg/dl)^a^	1.02	(1.01–1.03)	**0.002**	1.01	(1.002–1.03)	**0.017**
C-reactive protein (mg/dl)	1.06	(1.04–1.07)	**<0.001**	b		
Lactate dehydrogenase (U/L)^a^	1.02	(1.02–1.03)	**<0.001**	b		
Urea (mg/dl)^a^	1.07	(1.04–1.10)	**<0.001**	1.08	(1.03–1.13)	**0.003**

HR, hazard ratio; aHR, adjusted hazard ratio; CI, confidence interval.

The multivariable (adjusted) model composed by biochemical markers was generated using Cox regression and the nested model method with backward selection. The most parsimonious model was selected using the Wald test (p < 0.05) for a pool analysis of imputed datasets. Values in bold indicate significant associations (p < 0.05).

a Variables scaled/10 to better interpret the HR.

b Dynamic aHR estimations are illustrated in Fig. 2 for variables transform in restrictive cubic splines due to their lack of linearity.

**Table 4b:** Adjusted analysis of hematological markers associated with COVID-19 mortality.

	COVID-19 mortality – hematological markers											
	Non-adjusted Model			Model A			Model B			Model C		
	**HR**	**95% CI**	**p**	**aHR**	**95% CI**	**p**	**aHR**	**95% CI**	**p**	**aHR**	**95% CI**	**p**
Oxygen saturation (%)	**0.97**	(0.95–0.98)	**<0.001**	b			b			b		
Absolute neutrophil count x 10^3^/μl	1.08	(1.05–1.12)	**<0.001**	1.09	(1.05–1.13)	**<0.001**	1.09	(1.05–1.13)	**<0.001**	1.09	(1.05–1.13)	**<0.001**
Platelet count x 10^3^/μl^a^	0.98	(0.96–0.99)	**0.003**	0.98	(0.97–0.999)	**0.043**	0.98	(0.97–0.999)	**0.043**	0.98	(0.97–0.999)	**0.037**
Prothrombin time (s)	**1.03**	(1.001–1.06)	**0.044**	1.05	(1.01–1.08)	**0.007**	1.05	(1.01–1.08)	**0.007**			

HR, hazard ratio; aHR, adjusted hazard ratio; CI, confidence interval.

The multivariable (adjusted) models composed by hematological markers were generated using Cox regression and the nested model method with backward selection. Variables were specified according to the following criteria: Model A, modeling included all hematological variables; Model B, global modeling excluding variables expressing relative values; Model C, modeling including only complete blood count. The most parsimonious models were selected using the Wald test (p < 0.05) for a pool analysis of imputed datasets. Values in bold indicate significant associations (p < 0.05).

a Variables scaled/10 to better interpret the HR.

b Dynamic aHR estimations are illustrated in Fig. 2 for variables transform in restrictive cubic splines due to their lack of linearity.

### 3.6 Time-dependent ROC

**Fig. 3:**
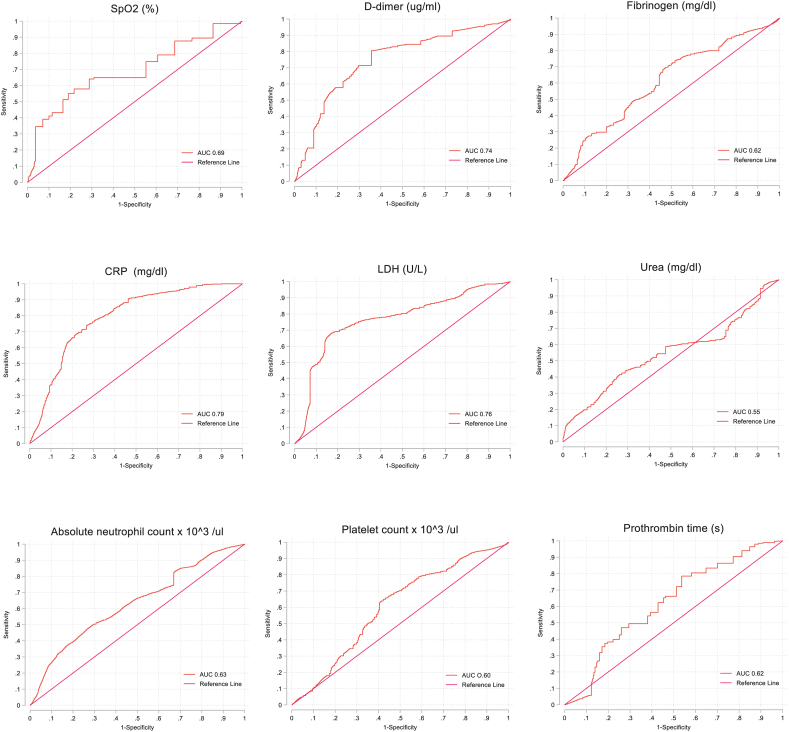
ROC curves of explanatory biomarkers at day 30. The individual performance of explanatory biomarkers was evaluated by a time-dependent receiver operating curve. None of them exceeded individually 0.79 of AUC.

At day 30, the individual explanatory variable with the largest AUC was CRP (0.79), with similar sensitivity and specificity. Other biomarkers displaying an AUC above 0.70 were D-dimer and LDH. On the other hand, hematological markers shaping models A, B and C, generally had low individual performances (AUC ∼0.60). The absolute neutrophil count best explained COVID-19 mortality at day 30. Complete information regarding the biomarkers’ prognostic capacity is detailed in Table 5. The different ROC curves are shown in Fig. 3.

**Table 5:** Sensitivity and specificity of the explanatory biomarkers.

	Cut-off	AUC	Sensitivity	Specificity	PPV	NPV
Oxygen saturation (%)	<89	0.69	64.10%	71.20%	70%	65%
D-dimer (ug/ml)	>1.98	0.74	80.60%	64.30%	71%	76%
Fibrinogen (mg/dl)	>487	0.62	68.30%	53.90%	61%	61%
C-reactive protein (mg/dl)	>9.90	0.79	74.10%	73.20%	75%	73%
Lactate dehydrogenase (U/L)	>346	0.76	68.80%	82.20%	80%	71%
Urea (mg/dl)	>45	0.55	58.80%	52.50%	57%	54%
Absolute neutrophil count x 10^3^/μl	>11.16	0.63	51.20%	69.20%	64%	57%
Platelet count x 10^3^/μl	<319	0.60	63.90%	58.70%	62%	60%
Prothrombin time (s)	<10.90	0.62	62.30%	57.20%	61%	59%

AUC, area under the curve; PPV, positive predictive value; NPV, negative predictive value.

The individual performance of explanatory biomarkers was evaluated by a time-dependent receiver operating curves (ROC) analysis (30 days), estimating the area under the curve (AUC) for each biomarker, sensitivity and specificity for an optimal cut-off point according to Youden index as implemented in the package sttroccurve.

## 4 Discussion

The current study aimed to identify blood markers associated with COVID-19 mortality during the first wave of the pandemic in Peru during 2020. This is also one of the few studies to evaluate a cohort severely compromised by COVID-19 at hospital admission [6,22], considering the absence of widespread vaccination and during the circulation of more virulent SARS-CoV-2 strains. Due to limited-resources, Peru and other low- and middle-income countries struggled to provide adequate healthcare to COVID-19 patients. In these settings, the identification of critical patients based on available platforms, such as the use of hematological and biochemical markers, is essential to determine various levels of hospital care.

The overall hospital mortality proportion was high compared to other countries [[23], [24], [25], [26]], but consistent with Peruvian studies; 46–49.59% in Lima [22,27] and 9% lower in the North Coast [6]. The elevated mortality proportion (51.60%) was due to the downgrading of hospital attention, which forced affected people to use in-house oxygen therapies and non-evidence-based drugs [5,6]. Delay in hospital management and increased disease duration influenced the high mortality rate compared to other contexts [28].

Oxygen saturation is the most important predictor of poor outcomes due its relation to alveolar deterioration, allowing the identification of cases with the greatest need for ventilatory support. The association of SpO2 <85% with a higher risk of death by COVID-19 was previously reported [12,22]. Dysregulation of immune response mediated by IL-6 induces an elevation of CRP [8] and ferritin levels >800 μg/L [29], observed in patients with severe or fatal COVID-19. CRP, a fast-increasing acute inflammatory indicator, exhibited in our study the highest levels identified in COVID-19 patients to date [12,24]. In our study, the role of fibrinogen predicted the risk of death in the study population. Similarly, high D-dimer levels produced during fibrin degradation suggest thrombosis and thrombolysis [8] and are associated with lower survival [12]. In our study, the results suggested that even patients who survived could be at risk of thrombosis on admission; however other features should be considered. LDH suggests vital organ injury, in which overregulation of the glycolytic pathway leads to a lack of oxygenation and multi-organ failure in severe COVID-19 patients [10]. However, in our cohort, LDH values remained within the reference ranges. On the other side, elevated urea indicates misfunction of kidney metabolism and is related to COVID-19 severity [30], alone or along with the albumin as a ratio [30,31]. In the study population, urea values were severely increased on admission among survivors and patients who died. Although patients seeking clinical care exhibited low SpO2 at hospital admission, many had access to portable oxygen during home care, which could have prevented organ injury before hospitalization.

Neutrophilia and Lymphopenia are caused by the dysfunction of the innate and adaptive immune response in severe COVID-19 patients, respectively [10,32]. In our study, survivors and fatal cases presented neutrophilia and lymphopenia, which was inconsistent with previous studies [[33], [34], [35]]. Our study population also presented very high NLR values, which have also been described before [11,36]. The lack of association of these values with the risk of death could be possibly because the entire population presented critical profiles on admission. Thrombocytopenia after inflammatory overregulation is controversially related to peripheral platelet consumption and thrombotic events among COVID-19 patients with poor outcomes [17,25,33,35,37]. A significant association has been reported between prolonged PT, a coagulopathy indicator, and an unfavorable prognosis, even when the PT values are within the reference range [35,38].

Thus, the evaluation of the prognostic potential for SpO2 was previously estimated as an AUC of 0.70 [12], which is compatible with our results (AUC 0.69). The reliability of CRP values is widely inconsistent, documenting good performances (AUC 0.86–0.92) in some studies [8,12] and weak performances (AUC 0.69) in others [33]. In our study, the AUC of CRP showed fair accuracy (0.79). Many reports have proposed the D-dimer cut-off to be somewhere between 0.67 and 2.03 μg/ml, with an AUC between 0.81 and 0.88 [8,12,39], showing better performance than in our study. Similarly, the AUC for LDH, ferritin, and NLR were estimated to be between 0.62 and 0.64, with only the absolute neutrophil count presenting an AUC of 0.53 [33]. Likewise, the AUC for urea was estimated at 0.69–0.78 [30,31]. Another study calculated the AUC for NLR at 0.77 [17], showing that this estimate can significantly differ from study to study. The platelet count in one study showed an AUC of 0.81 [17], presenting much better performance than in our study. Therefore, the efficiency of CRP, urea, LDH and D-dimer, the best-performing biomarkers, only achieved fair accuracy (none exceeded the accepted 80%). In relation to hematological markers, the individual diagnostic ability was not found to be good, with only the absolute neutrophil count showing a regular performance (AUC 0.63), albeit better than previous reports [33].

Models composed of combined covariates associated with the risk of mortality by COVID-19 aim to generate predictive tools capable of predicting poor COVID-19 outcomes. These models include different combinations of IL-6, CRP, D-dimer, absolute neutrophil count, and others, showing a generally good sensitivity and specificity (>90%) [8,35,40,41]. However, confirming the efficiency of these models requires an adequate modeling process and a comprehensive external validation [42]. The validation process reduces the overfitting of AUC estimation in training samplings, and thus, corrects the performance values. Other studies sought to generate models using only values of the CBC, the analysis of which is much more accessible, finding neutrophils, lymphocytes, and platelets to be explanatory variables for progression to unfavorable outcomes [37,43]. The multivariable models constructed in the present study did not report AUC values, as it was not possible to carry out external validation that would allow calculating the real AUC. Furthermore, internal validation of these models was beyond the aims of this study.

### 4.1 Limitations

The use of retrospective data remains a significant limitation that could impact data quality and introduce information bias. However, given the study context, a retrospective design was the only feasible option. Despite the challenges it entails, we anticipate that any registration errors are distributed uniformly. The inclusion of patients from a single site may induce selection bias; therefore, further research is needed to confirm these findings in diverse cohorts. Additionally, we identified the potential existence of Neyman bias, where the study population, more severely affected by COVID-19 might have been at higher risk of death. This inadvertently led to the exclusion of related biomarkers from the analysis. Undetermined markers, such as IL-6 (which presented the best AUC identified 0.931) [12], albumin, troponin, and others, were not measured. The inclusion of these markers is uncommon in routine clinical practice (troponin, for example, had 86% missing data in our study), and their incorporation does not align with the objective of identifying biomarkers available in resource-limited hospital settings.

As a result, we were unable to extract the medical indication for admission or determine actual ICU admissions. Local studies indicate that around 64% of patients may require ICU care, but only 3.30–10.20% are admitted [6,22,27]. In terms of statistical analysis, we employed a traditional variable selection strategy for multivariable modeling, which may overestimate associations. However, we opted for a backward method, considered preferable over the forward method [44], as our dataset included numerous candidate variables that could potentially serve as prognostic factors. Nonetheless, our data will be made available for independent groups to conduct alternative statistical analyses, contributing to a more comprehensive understanding of the role of biomarkers in poor COVID-19 outcomes within the scientific community.

Laboratory biomarkers are linked to the risk of death from COVID-19, and these parameters hold significant prognostic value. However, this association is primarily observed with biochemical markers, which are not routinely included in laboratory monitoring for COVID-19 patients in low- and middle-income hospitals. Hematological markers, being more cost-effective, monitor parameters uniformly for all inpatients. Every patient, regardless of their clinical condition, has equal priority for the determination of these parameters. While the AUC of each hematological marker associated with the risk of COVID-19 mortality did not exhibit high accuracy, combined covariates demonstrated a good fit. Moreover, the models constructed could be validated in other cohorts. Our study highlights that hematological markers can elucidate mortality from COVID-19 and are valuable in aiding clinical decision-making to prioritize critical care for COVID-19 patients. These tools, alongside other public health initiatives, will bolster a rapid response and facilitate preparedness strategies for the ongoing COVID-19 emergency and future pandemics.

## 5 Conclusion

Biomarkers associated with the risk of mortality were identified in the severe COVID-19 cohort. Multivariable models revealed that, in addition to oxygen saturation, hematological counts—such as the absolute neutrophil count, platelet count, and PT—assessed at hospital admission exhibited a good fit and held significant prognostic value in discriminating patients at risk of death from COVID-19. When considering individual prognostic performance, biochemical markers outperformed hematological parameters.

## Contributions

ST and JM designed the study. ST and JC collected the data. PSB, SM and JM design and adjusted the analysis strategy. SM and PS analyzed the data, interpreted it, and prepared figures and tables. SM drafted the manuscript. VF and JC commented on the final draft. JM, ST, MA and CD provided critical review and revised the manuscript. All authors read and approved the final manuscript.

## Funding source

The authors did not receive any funding for the work.

## Ethical approval

This study was approved by the Ethics and Research Committee of the Red Prestacional Lambayeque - Hospital Nacional Almanzor Aguinaga Asenjo, determination N° 026-CIEI-RPLAMB, and performed in accordance with the Declaration of Helsinki. The Ethics Committee waived the need for informed consent of each patient.

## Availability of data and materials

The database generated for the current study is included in the supplementary material, specifically in the “Additional information file 7” of the article.

## CRediT authorship contribution statement

**Stephanie Montero:** Writing – review & editing, Writing – original draft, Methodology, Investigation, Formal analysis, Conceptualization. **Jorge L. Maguiña:** Writing – review & editing, Validation, Supervision, Resources, Conceptualization. **Percy Soto-Becerra:** Writing – review & editing, Validation, Methodology, Investigation, Formal analysis, Conceptualization. **Virgilio E. Failoc-Rojas:** Writing – review & editing, Methodology, Investigation, Data curation. **Jorge Chira:** Writing – review & editing, Project administration, Data curation. **Moisés Apolaya-Segura:** Writing – review & editing, Supervision, Project administration, Investigation. **Cristian Díaz-Vélez:** Writing – review & editing, Supervision, Investigation, Funding acquisition. **Stalin Tello-Vera:** Writing – review & editing, Validation, Supervision, Project administration, Investigation, Data curation, Conceptualization.

## Declaration of competing interest

The authors declare that they have no known competing financial interests or personal relationships that could have appeared to influence the work reported in this paper.
