# Supplementary material for: Laboratory biomarkers associated with COVID-19 mortality among inpatients in a Peruvian referral hospital
Source: Heliyon. 2024 Feb 29;10(6):e27251. doi: 10.1016/j.heliyon.2024.e27251 (PMC10945112; doi:10.1016/j.heliyon.2024.e27251)
Supplement: Multimedia component 1 [file mmc1.docx]

**Additional file 1. Reference values and determination method of laboratory biomarkers**

| **#** | **Biomarker** | **Variable** | **Absolute reference values** | **Relative reference values** | **Method and measurement** | **Equipment** | **Calibration** |
| --- | --- | --- | --- | --- | --- | --- | --- |
|  |  |  |  |  |  |  |  |
| 1 | Hematological | White blood cell count | 4 - 10 x 10^3^ /ul | N/A | Impedance/Cytometric | MINDRAY BC – 6800 | N/A |
| 2 | Hematological | Lymphocyte count | 0.8 – 4 x 10^3^ /ul | 20 – 40 % | Impedance/Cytometric | MINDRAY BC – 6800 | N/A |
| 3 | Hematological | Neutrophil count | 2 – 7 x 10^3^ /ul | 50 – 70 % | Impedance/Cytometric | MINDRAY BC – 6800 | N/A |
| 4 | Hematological | Hemoglobin | 11 – 16 gr/dl | N/A | Photometric | MINDRAY BC – 6800 | N/A |
| 5 | Hematological | Hematocrit | 37 – 54 % | N/A | Deferred | MINDRAY BC – 6800 | N/A |
| 6 | Hematological | Platelet count | 100 – 300 x 10^3^ /ul | N/A | Impedance | MINDRAY BC – 6800 | N/A |
| 7 | Hematological | Mean platelet volume | 6.5 – 12 fl | N/A | Impedance | MINDRAY BC – 6800 | N/A |
| 8 | Hematological | Platelet distribution width | 15 – 17 | N/A | Deferred | MINDRAY BC – 6800 | N/A |
| 9 | Hematological | Platelet large cell ratio | 11 – 45 % | N/A | Impedance | MINDRAY BC – 6800 | N/A |
| 10 | Hematological | Plaquetocrit | 0.108 – 0.282 % | N/A | Impedance | MINDRAY BC – 6800 | N/A |
| 11 | Biochemical | Prothrombin time | 9.8 – 12.1 segundos | N/A | Photooptic/Plasma coagulation | BCS XP | N/A |
| 12 | Biochemical | Partial thromboplastin time activated | 27.6 - 37.4 seg | N/A | Photooptic/Plasma coagulation with phospholipids and surface activator* | SIEMENS BCS | PT-Multi calibrador |
| 13 | Biochemical | International Normalized Ratio for coagulation factors | 0.8 - 1.32 | N/A | Ratio PT test/PT normal, according to international sensitivity index* | SIEMENS BCS | PT-Multi calibrador |
| 14 | Biochemical | C-reactive protein | 0.5 mg/dl | N/A | Turbidimetric/antigen-antibody reaction/Tina quant | ROCHE/HITACHI – COBAS 6000 | Complete |
| 15 | Biochemical | D-dimer | 0.5 ug/ml | N/A | Turbidimetric/antigen-antibody reaction/Tina quant | BCS XP | Complete |
| 16 | Biochemical | Lactate dehydrogenase | 240 – 480 U/L | N/A | Photometric/enzimatic UV | ROCHE/HITACHI – COBAS 6000 | Blank |
| 17 | Biochemical | Fibrinogen | 500 mg/dl | N/A | Photooptic/Method of Claus modified | BCS XP | N/A |
| 18 | Biochemical | Ferritin | M: 30 – 400 ng/ml, W: 13 – 150 ng/ml | N/A | Photomultiplier/Sandwich test | ROCHE/HITACHI – COBAS 6000 | Two point |
| 19 | Biochemical | Urea | 10 – 50 mg/dl | N/A | Photometric/enzimatic UV/Talke and Schubert optimized | ROCHE/HITACHI – COBAS 6000 | Two point |
| 20 | Biochemical | Creatinine | M: 0.5 – 1.2 mg/dl; W: 0.4 – 1.1 mg/dl | N/A | Photometric/cinetic color/Jaffe, rate-blanked with compensation | ROCHE/HITACHI – COBAS 6000 | Two point |
| 21 | Biochemical | Glucose | 70 – 105 mg/dl | N/A | Photometric/enzimatic color/GOD-PAP modified | ROCHE/HITACHI – COBAS 6000 | Blank and two point |
| 22 | Biochemical | Aspartate aminotransferase | M: 37 U/L; W: 31 U/L | N/A | Photometric/enzimatic UV/IFCC standarized | ROCHE/HITACHI – COBAS 6000 | Blank |
| 23 | Biochemical | Alanine aminotransferase | M: 41 U/L; W: 31 U/L | N/A | Photometric/enzimatic UV/IFCC standarized | ROCHE/HITACHI – COBAS 6000 | Blank |
|  |  |  |  |  |  |  |  |
|  | |  |  |  |  |  |  |
| M,Men; W, women | |  |  |  |  |  |  |
| IFCC, International Federation of Clinical Chemistry and Laboratory Medicine | | | | | | | |
| *Test inlcudes the use of normal and pathologic control | | | | | | | |
