# Supplementary material for: Laboratory biomarkers associated with COVID-19 mortality among inpatients in a Peruvian referral hospital
Source: Heliyon. 2024 Feb 29;10(6):e27251. doi: 10.1016/j.heliyon.2024.e27251 (PMC10945112; doi:10.1016/j.heliyon.2024.e27251)
Supplement: Multimedia component 2 [file mmc2.docx]

**Additional file 2. Multiple imputation by chained equations**

**Methods**

We addressed missing data with multiple imputation by chained equations assuming missing at randomness. We created 20 imputed datasets and burnin runs of 10 iterations. Imputation model included all covariates evaluated in the modelling process, outcome and the Nelson-Aaleen estimator of cumulative hazard (van Buuren, 2018). All analyses were conducted using Stata software v.14 (Stata Corp. College Station, TX).

**Results**

At least one missing data was present in 18.60% (40/215) of overall patient data of variables of interest (SpO2, absolute lymphocyte count, fibrinogen, D-dimer, TP, aPTT, INR, LDH, CRP, urea, ferritin, ALT, AST, and creatinine).

**References**

van Buuren, S., 2018. Flexible imputation for missing data, Second. ed. Chapman & Hall/CRC.
