# Supplementary material for: Laboratory biomarkers associated with COVID-19 mortality among inpatients in a Peruvian referral hospital
Source: Heliyon. 2024 Feb 29;10(6):e27251. doi: 10.1016/j.heliyon.2024.e27251 (PMC10945112; doi:10.1016/j.heliyon.2024.e27251)
Supplement: Multimedia component 4 [file mmc4.docx]

**Additional file 4. Data distribution of case-complete and imputed datasets**

|  | **Total (complete-case) n = 215 (%)** | **Total (imputed) n = 4,515 (%)** |
| --- | --- | --- |
|  |  |  |
| Age (years) | 63.90±14.14 | 63.90±14.11 |
|  |  |  |
| Sex |  |  |
| Women | 61 (28.37) | 1,281 (28.37) |
| Men | 154 (71.63) | 3,234 (71.63) |
|  |  |  |
| Disease duration (days) * | 9.06±4.06 | 9.07±4.06 |
|  |  |  |
| Lenght of hospital stay (days) | 11 [6-18] | 11 [6-19] |
|  |  |  |
| Comorbidities |  |  |
| Obesity | 21 (9.77) | 441 (9.77) |
| Diabetes mellitus | 51 (23.72) | 1,071 (23.72) |
| Hypertension | 82 (38.14) | 1,722 (38.14) |
| Chronic kidney disease (CKD) | 21 (9.77) | 441 (9.77) |
| Other pulmonary disease | 21 (9.77) | 441 (9.77) |
|  |  |  |
| N° comorbidities |  |  |
| 0 | 78 (36.28) | 1,638 (36.28) |
| 1 | 89 (41.40) | 1,868 (41.40) |
| ≥2 | 48 (22.33) | 1,008 (22.33) |
|  |  |  |
| Oxygen saturation (%) * | 88 [82-92] | 88 [82-92] |
|  |  |  |
| Hemoglobin (g/dL) | 13.28±1.89 | 13.28±1.88 |
|  |  |  |
| Hematocrit (%) | 40 [37-43] | 40 [37-43] |
|  |  |  |
| White blood cell count x 10^3^ /ul | 11.71 [8.5-16.18] | 11.71 [8.5-16.18] |
|  |  |  |
| Relative lymphocyte count (%) | 6 [4-10] | 6 [4-10] |
|  |  |  |
| Absolute lymphocyte count x 10^3^ /ul | 0.71 [0.45-1.12] | 0.71 [0.45-1.12] |
|  |  |  |
| Relative neutrophil count (%) | 89 [84-93] | 89 [84-93] |
|  |  |  |
| Absolute neutrophil count x 10^3^ /ul | 10.30 [7.30-14.49] | 10.30 [7.30-14.49] |
|  |  |  |
| Neutrophil/Lynphocyte ratio | 14.5 [8.4-23.51] | 14.5 [8.4-23.51] |
|  |  |  |
| Platelet count x 10^3^ /ul | 322.11±120.10 | 322.11±119.83 |
|  |  |  |
| Mean platelet volume | 9.67±1.10 | 9.67±1.10 |
|  |  |  |
| Platelet distribution width | 16.3 [16.1-16.6] | 16.3 [16.1-16.6] |
|  |  |  |
| Plaquetocrit % | 0.3040±0.1049 | 0.3040±0.1046 |
|  |  |  |
| Platelet large cell ratio | 24.33±7.07 | 24.33±7.06 |
|  |  |  |
| Prothrombin time * | 11.1 [10.4-12.1] | 11.1 [10.4-12.1] |
|  |  |  |
| Partial thromboplastin time activated * | 1 [0.9-29.7] | 1 [0.9-30] |
|  |  |  |
| International Normalized Ratio for coagulation factors * | 26 [1-32] | 26 [1-32] |
|  |  |  |
| Atypic lymphocytes | 133 (61.86) | 2,772 (61.86) |
|  |  |  |
| Promyelocytes | 1 (0.47) | 21 (0.47) |
|  |  |  |
| Myelocytes | 48 (22.43) | 1,008 (22.43) |
|  |  |  |
| Metamyelocytes | 54 (25.23) | 1,134 (25.23) |
|  |  |  |
| Band form neutrophils | 123 (57.75) | 2,583 (57.75) |
|  |  |  |
| Fibrinogen (mg/dl) * | 519 [400-654] | 519 [400-654] |
|  |  |  |
| D-dimer (ug/ml) * | 2.3 [1.36-4.56] | 2.3 [1.36-4.56] |
|  |  |  |
| Lactate dehydrogenase (U/L) * | 350 [256-467] | 350 [256-467] |
|  |  |  |
| C-reactive protein (mg/dl) * | 10.7 [4.2-17.7] | 10.9 [4.2-17.7] |
|  |  |  |
| Urea (mg/dl) * | 40.85 [30.9-56.8] | 40.85 [30.9-56.8] |
|  |  |  |
| Ferritin (mg/dl) * | 1 330 [800-2 000] | 1 396 [800-2 000] |
|  |  |  |
| Aspartate aminotransferase (U/L) * | 32.65 [21.1-51] | 32.60 [21.1-51] |
|  |  |  |
| Alanine aminotransferase (U/L) * | 41 [23-69] | 40.9 [23-69] |
|  |  |  |
| Glucose (mg/dl) | 129 [105-171] | 129 [105-171] |
|  |  |  |
| Creatinine (mg/dl)* | 0.69 [0.55-0.92] | 0.69 [0.55-0.92] |
|  |  |  |
|  |  |  |
| Twenty imputed additional datasets were created during the multiple imputed chained equations process | | |
| Mean ± standard deviation (SD), Median [IQR] | | |
| *Variables with missing data: length of the disease = 2, SpO2 = 1, D-dimer = 6, fibrinogen = 9, LDH = 2, CRP = 13, urea = 1, ferritin = 24, ALT = 1, AST =1, creatinine = 5 | | |
