# Supplementary material for: Laboratory biomarkers associated with COVID-19 mortality among inpatients in a Peruvian referral hospital
Source: Heliyon. 2024 Feb 29;10(6):e27251. doi: 10.1016/j.heliyon.2024.e27251 (PMC10945112; doi:10.1016/j.heliyon.2024.e27251)
Supplement: Multimedia component 5 [file mmc5.docx]

**Additional file 5. Case-complete and imputed non-adjusted analysis of biomarkers associated to COVID-19 mortality**

|  | **COVID-19 mortality** | | | | | | | | | |
| --- | --- | --- | --- | --- | --- | --- | --- | --- | --- | --- |
|  | **HR (case-complete)** | | **95% CI** | | **p** | |  | **HR (imputed)** | **95% CI** | **p** |
|  |  | |  | |  | |  |  |  |  |
| Age (years) | **1.02** | | **(1.01 - 1.03)** | | **0.005** | |  | **1.02** | **(1.017 - 1.02)** | **<0.001** |
|  |  | |  | |  | |  |  |  |  |
| Sex |  | |  | |  | |  |  |  |  |
| Women | Ref | |  | |  | |  | Ref |  |  |
| Men | 1.00 | | (0.65 - 1.53) | | 0.997 | |  | 1.00 | (0.91 - 1.10) | 0.984 |
|  |  | |  | |  | |  |  |  |  |
| Duration (days) * | 1.03 | | (0.99 - 1.07) | | 0.118 | |  | 1.03 | (1.02 - 1.04) | **<0.001** |
|  |  | |  | |  | |  |  |  |  |
| Comorbidities |  | |  | |  | |  |  |  |  |
| Obesity | 1.65 | | (0.94 - 2.90) | | 0.081 | |  | 1.65 | (1.46 - 1.87) | **<0.001** |
| Diabetes mellitus | 1.01 | | (0.66 - 1.54) | | 0.968 | |  | 1.01 | (0.92 - 1.11) | 0.856 |
| Hypertension | 1.30 | | (0.89 - 1.90) | | 0.169 | |  | 1.30 | (1.20 - 1.41) | **<0.001** |
| Chronic kidney disease (CKD) | **1.97** | | **(1.17 - 3.31)** | | **0.010** | |  | **1.97** | **(1.76 - 2.21)** | **<0.001** |
| Other pulmonary disease | 0.87 | | (0.44 - 1.73) | | 0.700 | |  | 0.87 | (0.75 - 1.01) | 0.077 |
|  |  | |  | |  | |  |  |  |  |
| N° comorbidities |  | |  | |  | |  |  |  |  |
| 0 | Ref | |  | |  | |  | Ref |  |  |
| 1 | 0.93 | | (0.60 - 1.45) | | 0.757 | |  | 0.93 | (0.85 - 1.03) | 0.155 |
| ≥2 | 1.23 | | (0.77 - 1.99) | | 0.387 | |  | 1.23 | (1.11 - 1.37) | **<0.001** |
|  |  | |  | |  | |  |  |  |  |
| Oxygen saturation (%) * | **0.97** | | **(0.95 - 0.98)** | | **<0.001** | |  | **0.97** | **(0.97 - 1.00)** | **<0.001** |
|  |  | |  | |  | |  |  |  |  |
| Hemoglobin (g/dL) | 0.98 | | (0.90 - 1.08) | | 0.743 | |  | 0.98 | (0.90 - 1.08) | 0.133 |
|  |  | |  | |  | |  |  |  |  |
| Hematocrit (%) | 1.00 | | (0.97 - 1.04) | | 0.885 | |  | 1.00 | (0.995 - 1.01) | 0.506 |
|  |  | |  | |  | |  |  |  |  |
| White blood cell count x 10^3^ /ul | **1.07** | | **(1-04 - 1.11)** | | **<0.001** | |  | **1.07** | **(1.07 - 1.08)** | **<0.001** |
|  |  | |  | |  | |  |  |  |  |
| Relative lymphocyte count (%) | **0.94** | | **(0.89 - 0.98)** | | **0.004** | |  | **0.94** | **(0.93 - 0.94)** | **<0.001** |
|  |  | |  | |  | |  |  |  |  |
| Absolute lymphocyte count x 10^3^ /ul | 1.07 | | (0.75 - 1.51) | | 0.723 | |  | 1.07 | (0.99 - 1.15) | 0.105 |
|  |  | |  | |  | |  |  |  |  |
| Relative neutrophil count (%) | **1.04** | | **(1.01 - 1.07)** | | **0.008** | |  | **1.04** | **(1.03 - 1.05)** | **<0.001** |
|  |  | |  | |  | |  |  |  |  |
| Absolute neutrophil count x 10^3^ /ul | **1.08** | | **(1.05 - 1.12)** | | **<0.001** | |  | **1.08** | **(1.07 - 1.09)** | **<0.001** |
|  |  | |  | |  | |  |  |  |  |
| Neuthophil/Lynphocyte ratio | 1.01 | | (0.997 - 1.02) | | 0.160 | |  | 1.01 | (1.004 - 1.01) | **<0.001** |
|  |  | |  | |  | |  |  |  |  |
| Platelet count x 10^3^ /ul | **0.98** | | **(0.96 - 0.99)** | | **0.003** | |  | **0.98** | **(0.97 - 0.98)** | **<0.001** |
|  |  | |  | |  | |  |  | . |  |
| Mean platelet volume | 1.00 | | (0.84 - 1.20) | | 0.981 | |  | 1.00 | (0.96 - 1.04) | 0.912 |
|  |  | |  | |  | |  |  |  |  |
| Platelet distribution width | 0.99 | | (0.86 - 1.14) | | 0.923 | |  | 0.99 | (0.96 - 1.02) | 0.656 |
|  |  | |  | |  | |  |  |  |  |
| Plaquetocrit % | **0.06** | | **(0.01 - 0.34)** | | **0.002** | |  | **0.06** | **(0.04 - 0.08)** | **<0.001** |
|  |  | |  | |  | |  |  | . |  |
| Platelet large cell ratio | 1.00 | | (0.98 - 1.03) | | 0.728 | |  | 1.00 | (0.999 - 1.01) | 0.111 |
|  |  | |  | |  | |  |  |  |  |
| Prothrombin time * | **1.03** | | **(1.001 - 1.06)** | | **0.044** | |  | **1.03** | **(1.02 - 1.03)** | **<0.001** |
|  |  | |  | |  | |  |  |  |  |
| Partial thromboplastin time activated * | 1.00 | | (0.99 - 1.02) | | 0.471 | |  | 1.00 | (1.002 - 1.01) | **0.001** |
|  |  | |  | |  | |  |  |  |  |
| International Normalized Ratio for coagulation factors * | 1.00 | | (0.99 - 1.01) | | 0.547 | |  | 1.00 | (1.001 - 1.005) | **0.005** |
|  |  | |  | |  | |  |  |  |  |
| Fibrinogen (mg/dl) * | **1.02** | | **(1.001 - 1.03)** | | **0.003** | |  | **1.02** | **(1.01 - 1.02)** | **<0.001** |
|  |  | |  | |  | |  |  |  |  |
| D-dimer (ug/ml) * | **1.14** | | **(1.10 - 1.19)** | | **<0.001** | |  | **1.14** | **(1.13 - 1.14)** | **<0.001** |
|  |  | |  | |  | |  |  |  |  |
| Lactate dehydrogenase (U/L) * | **1.02** | | **(1.02 - 1.03)** | | **<0.001** | |  | **1.02** | **(1.021 - 1.025)** | **<0.001** |
|  |  | |  | |  | |  |  |  |  |
| C-reactive protein (mg/dl) * | **1.06** | | **(1.04 - 1.07)** | | **<0.001** | |  | **1.06** | **(1.05 - 1.06)** | **<0.001** |
|  |  | |  | |  | |  |  |  |  |
| Urea (mg/dl) * | **1.07** | | **(1.04 - 1.10)** | | **<0.001** | |  | **1.07** | **(1.06 - 1.08)** | **<0.001** |
|  |  | |  | |  | |  |  |  |  |
| Ferritin (mg/dl) * | **1.01** | | **(1.01 - 1.02)** | | **<0.001** | |  | **1.01** | **(1.012 - 1.013)** | **<0.001** |
|  |  | |  | |  | |  |  |  |  |
| Aspartate aminotransferase (U/L) * | 1.00 | | (0.996 - 1.01) | | 0.769 | |  | 1.00 | (0.9997 - 1.002) | 0.162 |
|  |  | |  | |  | |  |  |  |  |
| Alanine aminotransferase (U/L) * | 1.00 | | (0.99 - 1.000) | | 0.066 | |  | 1.00 | (0.995 - 0.997) | **<0.001** |
|  |  | |  | |  | |  |  |  |  |
| Glucose (mg/dl) | 1.00 | | (0.999 - 1.003) | | 0.243 | |  | 1.00 | (1.0007 - 1.0015) | **<0.001** |
|  |  | |  | |  | |  |  |  |  |
| Creatinine (mg/dl)* | **1.06** | | **(1.001 - 1.12)** | | **0.043** | |  | **1.06** | **(1.05 - 1.07)** | **<0.001** |
|  |  | |  | |  | |  |  |  |  |
|  |  | |  | |  | |  |  |  |  |
| Cox regression |  | |  | |  | |  |  |  |  |
| HR, Hazard ratio; CI, confidence interval |  | |  | |  | |  |  |  |  |
| *Variables with missing data |  | |  | |  | |  |  |  |  |
| **Variables scaled /10 to better interpret the HR: fibrinogen, LDH, urea, ferritin and platelet count | | | | | | | | | | |
|  | |  | |  | |  |  |  |  |  |
|  | |  | |  | |  |  |  |  |  |
|  | |  | |  | |  |  |  |  |  |
|  | |  | |  | |  |  |  |  |  |
|  | |  | |  | |  |  |  |  |  |
|  | |  | |  | |  |  |  |  |  |
|  | |  | |  | |  |  |  |  |  |
|  | |  | |  | |  |  |  |  |  |
|  | |  | |  | |  |  |  |  |  |
|  | |  | |  | |  |  |  |  |  |
|  | |  | |  | |  |  |  |  |  |
|  | |  | |  | |  |  |  |  |  |
|  | |  | |  | |  |  |  |  |  |
|  | |  | |  | |  |  |  |  |  |
