# Supplementary material for: Laboratory biomarkers associated with COVID-19 mortality among inpatients in a Peruvian referral hospital
Source: Heliyon. 2024 Feb 29;10(6):e27251. doi: 10.1016/j.heliyon.2024.e27251 (PMC10945112; doi:10.1016/j.heliyon.2024.e27251)
Supplement: Multimedia component 6 [file mmc6.docx]

**Additional file 6. Bivariate analysis of principal variables and observations with missing values**

|  | **Without missing n = 175** | **With missing n = 40** | **P**** |
| --- | --- | --- | --- |
|  |  |  |  |
| Outcome |  |  |  |
| Survivors | 86 (82.7) | 18 (17.3) | 0.636 |
| Deaths | 89 (80.2) | 22 (19.8) |  |
|  |  |  |  |

To address the assumption that missing at random is present in our database, we have evaluated the lack of association between a generated variable comprising missing values and the principal outcome. This result dismisses the possibility of missing completely at random. Although we cannot refuse that missing not at random could occur, the study design provides no reason to think that this phenomenon could take place and influence our estimations. Moreover, Additional files 4 and 5 demonstrate that the distribution of the variables and the strength of association were similar among case-complete and imputed datasets (van Buuren, 2018).

**References**

van Buuren, S., 2018. Flexible imputation for missing data, Second. ed. Chapman & Hall/CRC.
