# Supplementary material for: Laboratory biomarkers associated with COVID-19 mortality among inpatients in a Peruvian referral hospital
Source: Heliyon. 2024 Feb 29;10(6):e27251. doi: 10.1016/j.heliyon.2024.e27251 (PMC10945112; doi:10.1016/j.heliyon.2024.e27251)
Supplement: Multimedia component 8 [file mmc8.docx]

**Additional file 8. Characteristics, hematological count, and biochemical markers of the study population including Chi-square, median value, and T-statistic Student’s T**

**A**

|  | **Survivors n = 104 (%)** | **Deaths n = 111 (%)** | **Chi-square/Median value** | **T-statistic Student’s T** | **P ^b^** |
| --- | --- | --- | --- | --- | --- |
|  |  |  |  |  |  |
| Age (years) | 60.06±14.49 | 67.50±12.86 | NA | -3.9913 | **<0.001** |
|  |  |  |  |  |  |
| Sex |  |  |  |  |  |
| Women | 32 (52.46) | 29 (47.54) | 0.5696 | NA | 0.450 |
| Men | 72 (46.75) | 82 (53.25) |  |  |  |
|  |  |  |  |  |  |
| Disease duration (days) ^a^ | 8.55±3.76 | 9.54±4.30 |  |  | 0.078 |
|  |  |  |  |  |  |
| Length of hospital stay (days) | 14 [8-21] | 8 [5-14] | NA | -1.7717 | **<0.001** |
|  |  |  |  |  |  |
| Comorbidities |  |  |  |  |  |
| Obesity | 7 (33.33) | 14 (66.67) | 2.1077 | NA | 0.147 |
| Diabetes mellitus | 22 (43.14) | 29 (56.86) | 0.7337 | NA | 0.392 |
| Hypertension | 32 (39.02) | 50 (60.98) | 4.6380 | NA | **0.031** |
| Chronic kidney disease | 4 (19.05) | 17 (80.95) | 8.0138 | NA | **0.005** |
| Other pulmonary diseases | 12 (57.14) | 9 (42.86) | 0.7169 | NA | 0.397 |
|  |  |  |  |  |  |
| N° comorbidities |  |  |  |  |  |
| 0 | 40 (51.28) | 38 (48.72) | 5.7133 | NA | 0.057 |
| 1 | 48 (53.93) | 41 (46.07) |  |  |  |
| ≥2 | 16 (33.33) | 32 (66.67) |  |  |  |
|  |  |  |  |  |  |
| Oxygen saturation (%) | 90 [88-94] | 85 [79-90] | 34.5520 | NA | **<0.001** |
|  |  |  |  |  |  |
| Fibrinogen (mg/dl) ^a^ | 488.5 [372-640] | 567 [450-700] | 1.5742 | NA | 0.210 |
|  |  |  |  |  |  |
| D-dimer (ug/ml) ^a^ | 1.40 [0.90-2.42] | 4 [2.20-5] | 43.2048 | NA | **<0.001** |
|  |  |  |  |  |  |
| Lactate dehydrogenase (U/L) ^a^ | 273 [225-350] | 439.50 [345-526] | 49.8943 | NA | **<0.001** |
|  |  |  |  |  |  |
| C-reactive protein (mg/dl) ^a^ | 5 [1.50-11] | 15.80 [10.20-22.30] | 41.9174 | NA | **<0.001** |
|  |  |  |  |  |  |
| Urea (mg/dl) ^a^ | 35.90 [27.60-44.80] | 49.60 [33-66.40] | 17.9767 | NA | **<0.001** |
|  |  |  |  |  |  |
| Ferritin (mg/dl) ^a^ | 850 [689-1 300] | 1 952.50 [1 409-2 000] | 55.5532 | NA | **<0.001** |
|  |  |  |  |  |  |
| Aspartate aminotransferase (U/L) ^a^ | 32.70 [21-51] | 32.60 [22-51] | 0.0000 | NA | 1.000 |
|  |  |  |  |  |  |
| Alanine aminotransferase (U/L) ^a^ | 45 [24-81.30] | 37 [22-62] | 1.1979 | NA | 0.274 |
|  |  |  |  |  |  |
| Glucose level (mg/dl) | 123.50 [101.50-166] | 135 [106-175] | 1.0615 | NA | 0.303 |
|  |  |  |  |  |  |
| Creatinine level (mg/dl) ^a^ | 0.66 [0.51-0.82] | 0.73 [0.58-1.02] | 2.5649 | NA | 0.109 |
|  |  |  |  |  |  |
|  |  |  |  |  |  |
| Data is presented as n (%), mean ± standard deviation (SD), and median [IQR] | | | | | |
| ^a^ Missing data was identified in the following variables: length of the disease = 2, SpO2 = 1, D-dimer = 6, fibrinogen = 9, LDH = 2, CRP = 13, urea = 1, ferritin = 24, ALT = 1, AST =1, creatinine = 5 | | | | | |
| ^b^ Bivariate analysis was performed using Student’s-t and Median test for continuous variables with normal and non-normal distribution, respectively, and Chi-square for categorical variables | | | | | |

**B**

|  | **Survivors n = 104 (%)** | **Deaths n = 111 (%)** | **Chi-square/Median value** | **T-statistic Student’s T** | **P ^b^** |  |
| --- | --- | --- | --- | --- | --- | --- |
|  |  |  |  |  |  |  |
| Hemoglobin level (g/dL) | 13.27±1.83 | 13.28±1.95 | NA | -0.0561 | 0.955 |  |
|  |  |  |  |  |  |  |
| Hematocrit % | 40 [37-42.85] | 40 [37-43] | 0.0038 | NA | 0.951 |  |
|  |  |  |  |  |  |  |
| White blood cell count x 10^3^ /ul | 9.79 [7.41-12.69] | 13.90 [10.97-19.12] | 27.6300 | NA | **<0.001** |  |
|  |  |  |  |  |  |  |
| Relative lymphocyte count (%) | 7 [4-12] | 5 [3-8] | 7.6309 | NA | **0.006** |  |
|  |  |  |  |  |  |  |
| Absolute lymphocyte count x 10^3^ /ul | 0.69 [0.44-1.13] | 0.71 [0.45-1.11] | 0.3799 | NA | 0.538 |  |
|  |  |  |  |  |  |  |
| Relative neutrophil count (%) | 87 [80-93] | 91 [86-94] | 8.6500 | NA | **0.003** |  |
|  |  |  |  |  |  |  |
| Absolute neutrophil count x 10^3^ /ul | 8.42 [6.29-11.61] | 12.78 [9.39-16.44] | 19.6922 | NA | **<0.001** |  |
|  |  |  |  |  |  |  |
| Neutrophil/Lymphocyte ratio | 12.79 [7.09-23.12] | 15.35 [9.89-30.37] | 5.8169 | NA | **0.012** |  |
|  |  |  |  |  |  |  |
| Platelet count x 10^3^ /ul | 349.54±124.72 | 296.41±110.07 | NA | 3.3161 | **0.001** |  |
|  |  |  |  |  |  |  |
| Mean platelet volume | 9.68±1.16 | 9.67±1.04 | NA | 0.0815 | 0.935 |  |
|  |  |  |  |  |  |  |
| Platelet distribution width | 16.30 [16-16.50] | 16.40 [16.10-16.60] | 1.7770 | NA | 0.183 |  |
|  |  |  |  |  |  |  |
| Plaquetocrit % | 0.33±0.11 | 0.28±0.10 | NA | 3.4999 | **<0.001** |  |
|  |  |  |  |  |  |  |
| Platelet large cell ratio % | 24.08±7.31 | 24.56±6.87 | NA | -0.4979 | 0.619 |  |
|  |  |  |  |  |  |  |
| Prothrombin time ^a^ | 10.85 [10.20-11.50] | 11.45 [10.60-12.40] | 7.5515 | NA | **0.006** |  |
|  |  |  |  |  |  |  |
| Partial thromboplastin time activated ^a^ | 1 [0.90-29.50] | 1 [0.90-29.70] | 0.7213 | NA | 0.396 |  |
|  |  |  |  |  |  |  |
| International normalized ratio for coagulation factors ^a^ | 24.50 [0.90-30] | 27.60 [1-34.40] | 2.0762 | NA | 0.150 |  |
|  |  |  |  |  |  |  |
| Atypical lymphocytes | 60 (45.45) | 72 (54.55) | 1.3628 | NA | 0.243 |  |
|  |  |  |  |  |  |  |
| Promyelocytes | 0 (0) | 1 (100) | NA | NA | NA |  |
|  |  |  |  |  |  |  |
| Myelocytes | 26 (54.17) | 22 (45.83) | 0.7681 | NA | 0.381 |  |
|  |  |  |  |  |  |  |
| Metamyelocytes | 28 (51.85) | 26 (48.15) | 0.3061 | NA | 0.580 |  |
|  |  |  |  |  |  |  |
| Band form neutrophils | 53 (43.09) | 70 (56.91) | 3.8343 | NA | 0.050 |  |
|  |  |  |  |  |  |  |
|  |  |  |  |  |  |  |
| Data is presented as n (%), mean ± standard deviation (SD), and median [IQR] | | | | | | |
| ^a^ Missing data was identified in the following variables: PT = 1, aPTT = 1, INR = 2, promyelocytes = 1, myelocytes = 1, metamyelocytes = 1, band form neutrophils =2 | | | | | | |
| ^b^ Bivariate analysis was performed using Student’s-t and Median test for continuous variables with normal and non-normal distribution, respectively, and Chi-square for categorical variables | | | | | | |
